# Effectiveness and Safety of Ustekinumab for Ulcerative Colitis: A Brazilian Multicentric Observational Study

**DOI:** 10.1093/crocol/otae023

**Published:** 2024-04-11

**Authors:** Rogério Serafim Parra, Júlio Maria Fonseca Chebli, Matheus Freitas Cardoso de Azevedo, Liliana Andrade Chebli, Gilmara Pandolfo Zabot, Ornella Sari Cassol, Renata de Sá Brito Fróes, Genoile Oliveira Santana, Márcio Lubini, Daniela Oliveira Magro, Marcello Imbrizi, Antonio Carlos da Silva Moraes, Fabio Vieira Teixeira, Antonio José Tiburcio Alves Junior, Newton Luiz Tricarico Gasparetti Junior, Sandro da Costa Ferreira, Natália Sousa Freitas Queiroz, Paulo Gustavo Kotze, Omar Féres

**Affiliations:** Department of Surgery and Anatomy, Ribeirão Preto Medical School, University of São Paulo, Ribeirão Preto, São Paulo, Brazil; Division of Gastroenterology, Department of Medicine, Inflammatory Bowel Disease Center, Federal University of Juiz de Fora, Juiz de Fora, Minas Gerais, Brazil; Department of Gastroenterology, University of São Paulo School of Medicine, São Paulo, Brazil; Division of Gastroenterology, Department of Medicine, Inflammatory Bowel Disease Center, Federal University of Juiz de Fora, Juiz de Fora, Minas Gerais, Brazil; Department of Colon and Rectum Surgery, Moinhos de Vento Hospital, Feevale University, Porto Alegre, Brazil; Department of Colorectal Surgery, Hospital de Clínicas de Passo Fundo, Atitus Medical School, Rio Grande do Sul, Brazil; Department of Gastroenterology and Endoscopy, Gastromed, Rio de Janeiro, Brazil; Department of Life Sciences, State University of Bahia, Salvador, Bahia, Brazil; Department of Surgery, Passo Fundo University, Rio Grande do Sul, Brazil; Department of Surgery, State University of Campinas (UNICAMP), Campinas, São Paulo, Brazil; Department of Surgery, State University of Campinas (UNICAMP), Campinas, São Paulo, Brazil; Internal Medicine Department, Hospital Copa D´Or, Rio de Janeiro, Brazil; Department of Gastroenterology, Gastrosaúde Clinic, Marilia, São Paulo, Brazil; Department of Surgery, PUC-Campinas Medical School, PUC-Campinas University, Campinas, São Paulo, Brazil; Department of Surgery, Hospital Vivalle Rede Dor, São José dos Campos, São Paulo, Brazil; Department of Medicine, Ribeirão Preto Medical School, University of São Paulo, Ribeirão Preto, São Paulo, Brazil; Health Sciences Graduate Program, Pontifícia Universidade Católica do Paraná (PUCPR), Curitiba, Brazil; IBD Center, Santa Cruz Hospital, Curitiba, Brazil; Internal Medicine Department, Hospital Copa D´Or, Rio de Janeiro, Brazil; Health Sciences Graduate Program, Pontifícia Universidade Católica do Paraná (PUCPR), Curitiba, Brazil; Department of Colorectal Surgery, Hospital de Clínicas de Passo Fundo, Atitus Medical School, Rio Grande do Sul, Brazil; Department of Surgery and Anatomy, Ribeirão Preto Medical School, University of São Paulo, Ribeirão Preto, São Paulo, Brazil

**Keywords:** biological therapy, ulcerative colitis, inflammatory bowel disease, ustekinumab, real world

## Abstract

**Background:**

Real-world data on the effectiveness and safety of ustekinumab (UST) in ulcerative colitis (UC) are lacking in Latin America. In this study, we aimed to describe the effectiveness and safety of UST in a real-world multicenter cohort of Brazilian patients with UC.

**Methods:**

We conducted a multicenter retrospective observational cohort study, including patients with moderate-to-severe UC (total Mayo score 6–12, with an endoscopic subscore of 2 or 3) who received UST. The co-primary endpoints were clinical remission, defined as a total Mayo score ≤2 at 1 year, with a combined rectal bleeding and stool frequency subscore of ≤1, and endoscopic remission (endoscopic Mayo subscore of 0) within 1 year from baseline. Secondary endpoints included clinical response between weeks 12 and 16, endoscopic response within 1 year of starting UST, steroid-free clinical remission at week 52, and biochemical remission at week 52. We also evaluated UST treatment persistence and safety.

**Results:**

A total of 50 patients were included (female, *n* = 36, 72.0%), with a median disease duration of 9.2 years (1–27). Most patients had extensive colitis (*n* = 38, 76.0%), and 43 (86.0%) were steroid dependent at baseline. Forty patients (80.0%) were previously exposed to biologics (anti-TNF drugs, *n* = 31; vedolizumab [VDZ], *n* = 27). The co-primary endpoints of clinical remission at 1 year and endoscopic remission within 1 year were achieved by 50.0% and 36.0% of patients, respectively. Clinical response at weeks 12–16 was 56.0%, and endoscopic response, steroid-free clinical remission, and biochemical remission at week 52 were 68.0%, 46.5%, and 50.0%, respectively. The UST treatment persistence rate at 24 months was 73.7%. During the follow-up, 10 patients (20.0%) were hospitalized, mostly due to disease progression, and 3 patients required colectomy. Nine patients (18.0%) discontinued the drug mainly due to a lack of effectiveness. Twenty-seven adverse events (AEs) were reported, 16 of which were considered as serious AEs.

**Conclusions:**

In this real-world cohort of difficult-to-treat UC patients, UST was associated with improvements in clinical, biochemical, and endoscopic outcomes. The safety profile was favorable, consistent with the known profile of UST.

## Graphical Abstract

**Graphical Abstract:**
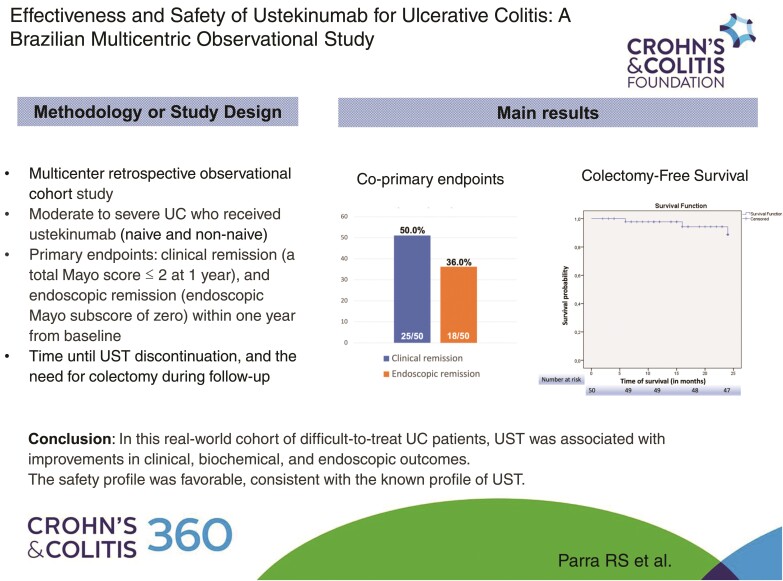

## Introduction

Ulcerative colitis (UC) is a chronic, progressive, and idiopathic immune-mediated disorder that affects the colon, with an increasing incidence and prevalence in recent years.^1^ The disease can have a negative impact on the quality of life, impair work productivity, and if not well controlled can lead to higher rates of hospitalization and the need for colectomy.^2–4^ Furthermore, despite medical advances, approximately 1 in 6 UC patients undergo colectomy within 10 years of diagnosis.^5^

Over the last 2 decades, several measures have improved the clinical outcomes of affected patients, including early diagnosis, the definition of more objective therapeutic targets, disease monitoring, and increased use of biologic agents.^5^ Anti-TNF agents were the first biologics used in the treatment of inflammatory bowel diseases, and their widespread use may have an impact on reducing overall colectomy rates in patients with UC.^3^

In Brazil, over the last 5 years, non-TNF-α inhibitors and small molecules have become increasingly available for the treatment of moderate-to-severe UC. However, despite the approval of these drugs, access to some biologics, such as ustekinumab (UST), is still restricted.^6,7^

UST is a human IgG1 monoclonal antibody that targets the p40 subunit of interleukin (IL)-12 and IL-23 for the treatment of moderately to severely active UC in patients who have had an inadequate response, loss of response, or were intolerant to either conventional therapy or a biologic or have medical contraindications to such therapies.^8^

Data from pivotal studies showed that UST is effective and safe in the treatment of moderate-to-severe UC, with low rates of side effects and high rates of remission through 4 years.^9^ However, it must be considered that the study populations in randomized controlled clinical trials do not faithfully represent the IBD population seen in clinical practice, mainly due to their stringent selection criteria, which limits the generalization of their results.^10^ Therefore, several studies have reported real-world experiences on the effectiveness and safety of UST in UC in recent years.^11–16^ Even so, most of these data come from Europe and North America, and there are still no data available from Latin America, to date, specifically in Brazil. Thus, in this study, we aimed to describe the effectiveness and safety of UST in a real-world multicenter cohort of Brazilian patients with UC.

## Methods

### Study Design and Population

This was an observational, retrospective multicenter study, including adult patients (≥18 years at the start of UST), with moderate-to-severe UC (total Mayo score of 6–12, with an endoscopic subscore of 2 or 3 as defined by endoscopic assessment within 3 months before starting UST therapy), who received at least a single dose of UST in 6 IBD centers and 6 private IBD clinics in Brazil. We only included patients who received the labeled dose of UST (6 mg/kg intravenous, followed by 90 mg subcutaneously every 8 weeks thereafter). Patients who received UST for indications other than UC (Crohn’s disease [CD], undetermined colitis), or who were in remission or had mild activity at baseline (total Mayo score of 0–5, or endoscopic subscore of 0–1) were not included. We also excluded patients with previous colectomy (partial or total colectomy, or ileoanal pouch) and those with missing available data.

### Data Collection and Ethical Approval

Patients were identified at each site through electronic medical record searches in all participating IBD centers. Patient demographic and clinical data were collected through a comprehensive review of their electronic medical records. Data were remotely monitored by the coordinating site to assess data quality. The following baseline characteristics were collected: sex, age at inclusion, age at diagnosis, disease duration, disease location (proctitis, left-sided colitis, or pancolitis) according to the Montreal classification,^17^ total Mayo score, endoscopic subscore, current smoking status, steroid-dependent status, biomarkers (C-reactive protein [CRP], fecal calprotectin [FC]), albumin, and hemoglobin levels, presence of extraintestinal manifestations (EIM), or associated immune-mediated inflammatory diseases. We also evaluated previous and current UC treatments (including immunomodulators such as methotrexate [MTX], azathioprine [AZA], or 6-mercaptopurine [6-MP], steroids, anti-TNF therapy, or other biologics, such as anti-integrin vedolizumab, and Janus kinase [JAK] inhibitors). Additionally, we included information regarding adverse events (AEs) during UST treatment, UC-related hospitalization, need for colectomy during UST treatment, primary nonresponse (PNR), secondary loss of response, reasons for drug discontinuation, and the need for dose optimization during maintenance therapy with UST. Dose optimization of UST was performed according to the physician’s discretion.

Data were included if collected within 4 weeks of each specified time point, except for the endoscopic reassessment after starting UST therapy, which was performed between weeks 26 and 52 at all participating centers. The study was approved by the ethics committee. All involved centers had Institutional Review Board approval. All procedures were conducted in accordance with the 1964 Declaration of Helsinki and its later amendments or comparable ethical standards.

### Definitions and Study Effectiveness Endpoints

The co-primary endpoints were clinical remission, defined as a total Mayo score ≤2 at 1 year, with a combined rectal bleeding and stool frequency subscore of ≤1, and endoscopic remission (ie, endoscopic Mayo subscore of 0) within 1 year of starting UST therapy. Secondary endpoints included clinical response, defined as a decrease of at least 3 points in the partial Mayo score between weeks 12 and 16, steroid-free clinical remission at week 52, biochemical response (a decrease of >50% in CRP and/or FC levels between weeks 12 and 16 in patients with a CRP >5 mg/L or FC >250 µg/g at baseline), biochemical remission (a CRP <5 mg/L and FC <150 µg/g at week 52 in patients with a CRP >5 mg/L and FC >250 µg/g at baseline), endoscopic response (endoscopic Mayo subscore of 0 or 1) within 1 year of starting UST therapy, and UST treatment persistence.

Primary nonresponse (PNR) was defined as the absence of clinical improvement within 16 weeks, leading to drug discontinuation. Secondary loss of response was defined as the recurrence of symptoms attributable to UC with a total Mayo score >6 and objective signs of inflammation detected by endoscopy, CRP >5 mg/L and/or FC >250 µg/g after responding to the drug during induction therapy. Steroid-free clinical remission was defined as the complete tapering of steroids in patients at 1 year in patients maintaining clinical remission, with no repeated steroid prescription within 4 weeks of tapering. Treatment persistence was defined as the duration of time from initiation to the last follow-up visit, discontinuation of UST, or switching to another therapy. The follow-up for treatment persistence analysis was limited to 24 months, as the number of patients treated with UST beyond this period was restricted.

### Safety

Safety outcomes included any reported infusion reactions, serious or nonserious infections, and any serious or non-SAEs. Adverse events were considered serious when they resulted in the discontinuation of UST, hospitalization, persistent/permanent or significant disability, death, or as deemed by the attending physician at the time of occurrence. Infections were deemed serious when intravenous antibiotics were required or when they led to the discontinuation of UST, hospitalization, permanent or significant disability, or death. We collected AE data throughout the follow-up period while patients were on treatment with UST. Reasons for drug discontinuation included a lack of primary response, surgery for UC, secondary loss of response to UST despite dose escalation, or serious adverse events (SAEs) that would necessitate discontinuing the drug. All patients who received at least 1 dose of UST were included in the safety analysis.

### Statistical Analysis

Categorical variables were presented as proportions and compared using chi-square or Fisher’s exact tests, as appropriate. Continuous variables were summarized using mean values, standard deviation (SD), median, and interquartile ranges [IQR]. Kaplan–Meier curves were generated for time-to-event data (time until UST discontinuation, in months), and the need for colectomy during follow-up. Data were reported using nonresponder imputation (NRI). Thus, patients who prematurely discontinued the study or had missing data were considered nonresponders in the statistical analyses for clinical, biochemical, and/or endoscopic response/remission. We utilized IBM SPSS Statistics for Windows, version 20.0 (IBM Corp). The significance level adopted for the statistical tests was 5%.

## Results

### Baseline Characteristics

A total of 56 patients aged ≥18 years with UC who received at least 1 dose of UST were identified through electronic medical record searches. However, 6 (10.7%) patients were excluded from the analysis due to previous colectomy (*n* = 4) and incomplete data in medical charts (*n* = 2), resulting in an overall study population of 50 patients. The mean duration of UST therapy was 19.2 months (SD 14.2 months; range 2–72 months). Demographics and clinical characteristics at baseline are presented in Table 1. In summary, most patients were female (*n* = 36, 72.0%), with a mean age of 42.8 years (range 21–72) and a mean disease duration of 9.2 years (range 1–27). Most patients had anemia (*n* = 27, 54.0%), and 10 patients (20.0%) presented with an extraintestinal manifestation (EIM), with half of them (*n* = 5, 10.0%) having concomitant psoriasis or psoriatic arthritis. One patient was an active smoker at baseline. Mean albumin serum levels were 3.66 g/dL (range 1.8–4.6 g/dL; *n* = 4 missing data), and 17 patients (36.9%, *n* = 17/46) had levels below 3.5 mg/L.

**Table 1.** Baseline clinical and demographic characteristics of ustekinumab-treated patients with ulcerative colitis (*n* = 50).

Characteristics	Results
Sex, female, *n* (%)	36 (72.0)
Mean age, years (IQR)	42.8 (13–72)
Mean disease duration, years (IQR)	9.2 (1–27)
Current smoker, *n* (%)	1 (2.0)
Anemia, n (%)	27 (54.0)
Extraintestinal manifestations, *n* (%)	10 (20.0)
Psoriasis/ psoriatic arthritis, *n* (%)	5 (10.0)
Disease extent, *n* (%)	
Proctitis	1 (2.0)
Left-sided colitis	11 (22.0)
Extensive colitis	38 (76.0)
Increased biomarkers^a^, *n* (%)	47 (94.0)
Mean C-reactive protein, mg/L (IQR)	15.3 (0.28–54.1)
Mean fecal calprotectin, µg/g (IQR)	2061.6 (403–6700)
Mean albumin serum levels, g/dL (IQR)^b^	3.7 (1.8–4.6)
Serum albumin <3.5 g/dL, *n* (%)	17 (36.9)
Total Mayo score, mean (IQR)	9.5 (6–12)
Endoscopic subscore 2 (Moderate disease), *n* (%)	14 (28.0)
Endoscopic subscore 3 (Severe disease), *n* (%)	36 (72.0)
Concomitant use of corticosteroids, *n* (%)	43 (86.0)
Previous exposure to immunomodulators^c^	42 (84.0)
Concomitant use of immunomodulators, *n* (%)^c^	7 (14.0)
Previous advanced therapy^d^	40 (80.0)
Number of previous biologics or JAK inhibitor, *n* (%)	
0	10 (20.0)
1	21 (42.0)
2	12 (24.0)
3	7 (14.0)
Previous exposure to specific advanced therapies, *n* (%)	
Anti-TNF	31 (62.0)
Infliximab	29 (58.0)
Adalimumab	8 (16.0)
Golimumab	1 (2.0)
Vedolizumab	27 (54.0)
Tofacitinib	1 (2.0)

Abbreviations: IQR, interquartile range; TNF, tumor necrosis factor; UST, ustekinumab.

^a^C-reactive protein (CRP) or fecal calprotectin (FC) higher than 5.0 mg/L or 150 µg/g, respectively. No missing data regarding CRP levels. Seven patients have no FC levels available at baseline.

^b^Missing data in 4 patients at baseline.

^c^Immunomodulators were defined as thiopurines (azathioprine or 6-mercaptopurine) and methotrexate.

^d^Advanced therapies included anti-TNF agents, vedolizumab, and tofacitinib.

Most patients had extensive colitis (*n* = 38, 76.0%), and 43 (86.0%) were steroid dependent at baseline. Mean CRP was 15.3 mg/L (range 0.28–54.1 mg/L), and it was elevated (CRP >5.0 mg/L) in 38 patients (76.0%); mean FC was 2061.6 µg/g (range 403–6700), and it was elevated (FC >150 µg/g) in all patients (100%, *n* = 43; missing data in 7 patients at baseline). Overall, 47 patients (94.0%) had elevations in CRP and/or FC at baseline. The mean total Mayo score at baseline was 9.52 (range 6–12), and the endoscopic subscore at baseline was 2 (*n* = 14, 28.0%) or 3 (*n* = 36, 72.0%).

Forty-two patients (84.0%) had prior exposure to immunomodulators (thiopurines [azathioprine or 6-mercaptopurine] or methotrexate). Ten patients (20.0%) who initiated UST were naive to advanced therapies (biologics and/or JAK inhibitors). Forty patients (80.0%) had previous exposure to advanced therapies. In total, 30 patients had prior exposure to at least 1 anti-TNF drug (infliximab, *n* = 29; adalimumab, *n* = 7; golimumab, *n* = 1), 27 patients had previous exposure to vedolizumab, and 1 patient was exposed to tofacitinib. Concerning the number of previous advanced therapies, 21 patients had 1 prior biologic (infliximab, *n* = 11; vedolizumab, *n* = 9; adalimumab, *n* = 1); 12 patients had 2 prior biologics (infliximab and vedolizumab, *n* = 10; infliximab and adalimumab, *n* = 1; adalimumab and vedolizumab, *n* = 1); and 7 patients had 3 prior biologics and/or JAK inhibitors (infliximab, adalimumab, and vedolizumab, *n* = 5; vedolizumab, infliximab, and tofacitinib, *n* = 1; vedolizumab, infliximab, and golimumab, *n* = 1).

### Clinical, Biochemical, and Endoscopic Outcomes

**Figure 1.**
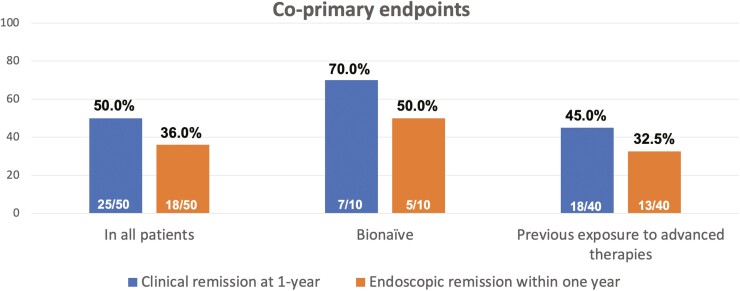
Clinical remission at 1 year and endoscopic remission within 1 year of ustekinumab treatment.

The primary endpoint of clinical remission at 1 year was achieved by 50.0% of patients (25/50), while endoscopic remission within 1 year [median (IQR): 30 (26–48) weeks] was observed in 18/50 (36.0%) patients. Additionally, the rates of clinical remission at 1 year and endoscopic remission within 1 year in bio-naïve patients were 70% (7/10) and 50% (5/10), respectively. Conversely, 45% (18/40) of bio-exposed patients achieved clinical remission, and 32.5% (13/40) achieved endoscopic remission at this time. These results are illustrated in Figure 1.

**Figure 2.**
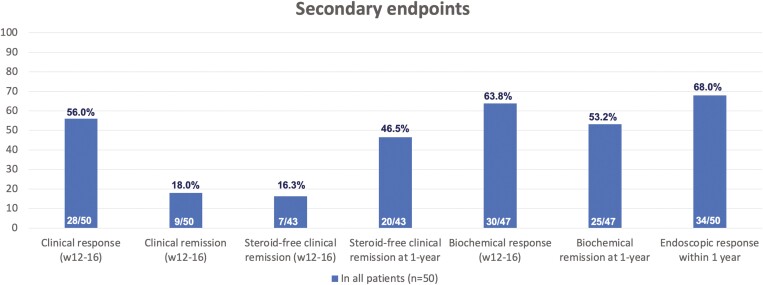
Clinical response and remission after ustekinumab induction (weeks 12–16), steroid-free clinical remission at 1 year, biochemical response after induction (weeks 12–16), biochemical remission at 1 year, and endoscopic response within 1 year.

Clinical response and clinical remission at weeks 12–16 were 56.0% and 18.0%, respectively. Steroid-free clinical remission was observed in 16.3% (*n* = 7/43) of patients at weeks 12–16. Endoscopic response was achieved in 68.0% of patients, and steroid-free clinical remission was observed in 46.5% (*n* = 20/43) at 1 year. Biochemical response at weeks 12–16 was 63.8% (*n* = 30/47), and biochemical remission at 1 year was 53.2% (*n* = 25/47). These results are shown in Figure 2.

**Figure 3.**
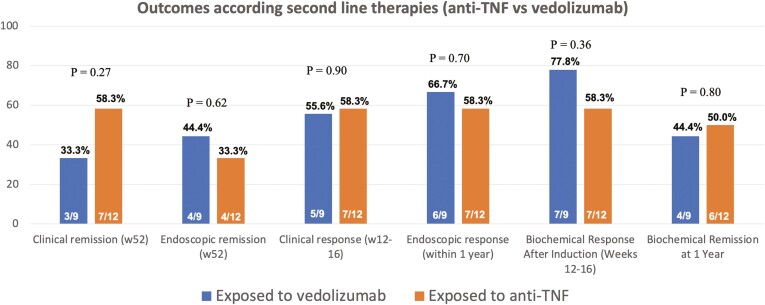
Clinical, biochemical, and endoscopic outcomes in patients with ulcerative colitis using ustekinumab as second-line therapy after vedolizumab or anti-TNF use.

Moreover, we conducted an analysis of outcomes based on the use of UST as a second-line therapy following the failure of vedolizumab (*n* = 9) or anti-TNFs (*n* = 12). No significant differences were observed in clinical remission rates at 52 weeks (58.3% vs. 33.3%, *P* = .27), endoscopic remission within 1 year (33.3% vs. 44.4%, *P* = .62), clinical response at weeks 12–16 (58.3% vs. 55.6%, *P* = .90), endoscopic response within 1 year (58.3% vs. 66.7%, *P* = .70), biochemical response at weeks 12–16 (58.3% vs. 77.8%, *P* = .36), and biochemical remission at 1 year (50.0% vs. 44.4%, *P* = .80). These findings are illustrated in Figure 3.

### Treatment Persistence, Optimization, and Colectomy-Free Survival

Eleven patients (22.0%) required dose escalation of UST therapy to 4 weekly. The proportion of patients with UC who regained biological and/or clinical response after increasing the dose to every 4 weeks (Q4W) was 54.5% (*n* = 6). Out of the 50 patients who started on UST, 7 patients (14.0%) were considered as PNR, while 9 (18.0%) discontinued the drug mainly due to a lack of effectiveness.

**Figure 4.**
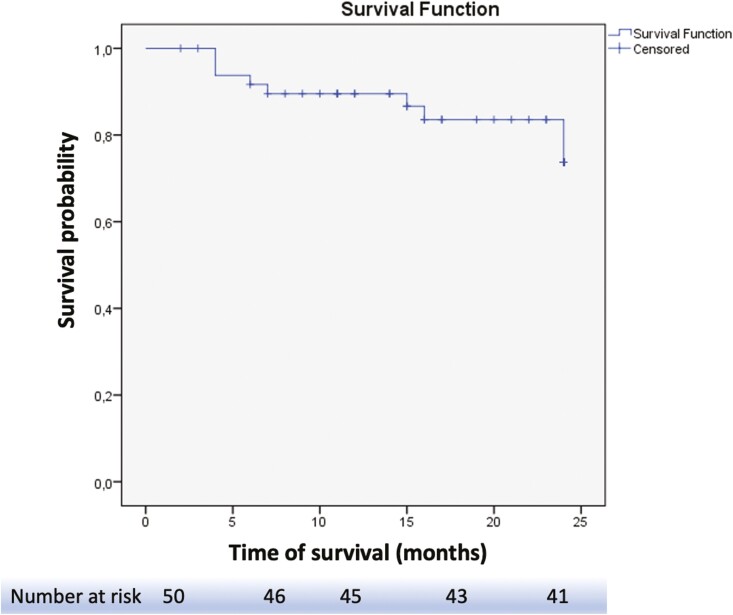
Kaplan–Meier curve for ustekinumab persistence in the whole cohort of patients with ulcerative colitis over the 24 months of follow-up.

The Kaplan–Meier curve analysis in Figure 4 illustrates the probability of UST treatment continuation. More precisely, the UST treatment persistence rates were as follows: 91.7% at 6 months, 89.5% at 12 months, 83.6% at 18 months, and 73.1% at 24 months.

**Figure 5.**
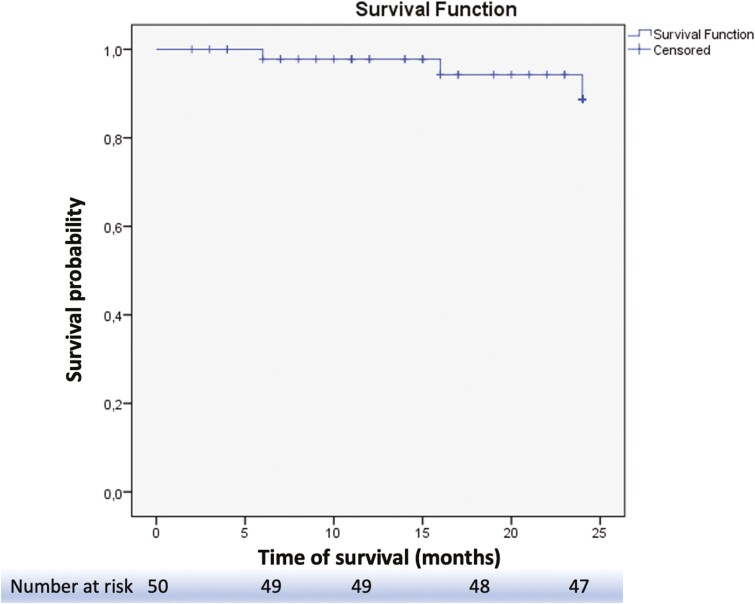
Kaplan–Meier curve showing colectomy-free survival during the follow-up of patients with ulcerative colitis under ustekinumab treatment.

We assessed colectomy-free survival at 24 months since there were few patients with follow-up data beyond this period. During the follow-up, 3 patients (6.0%) underwent colectomy. Figure 5 displays the Kaplan–Meier curve for colectomy-free survival. Specifically, the colectomy-free survival rates were 97.8% at 12 months and 88.7% at 24 months.

### Safety Outcomes

During the follow-up, 10 patients (20.0%) were hospitalized. Overall, 26 AEs were reported, of which 15 were considered SAEs. Hospitalization due to disease worsening was the most common SAE, reported in 9 (18.0%) patients, of whom 3 (6.0%) required a colectomy. One patient was diagnosed with prostate cancer at week 12 of UST treatment. Another patient developed a cutaneous rash 24 hours after the first subcutaneous dose, requiring hospitalization and withdrawal of UST therapy. One patient experienced a severe headache necessitating hospitalization after UST induction, and during hospitalization, she was diagnosed with cavernous sinus thrombosis. One patient had pulmonary embolism, likely related to UC activity. There were no reported deaths in this study. These results are summarized in Table 2.

**Table 2.** Safety events with ustekinumab treatment during the follow-up.

Adverse events/serious adverse events	*N* = 27
Deep vein thrombosis^a^	1
Headache	2
Constipation	1
Arthralgia	1
Lower urinary tract infection	2
Mild COVID-19	3
Serious adverse events	16
Hospitalization due to disease progression	9
Prostatic cancer^b^	1
Pulmonary embolism^c^	1
Cavernous sinus thrombosis^c^	1
Cutaneous rash^d^	1
Colectomy	3

^a^One episode of deep vein thrombosis associated with COVID-19 infection.

^b^This event is probably not related to ustekinumab.

^c^There was 1 case of pulmonary embolism associated with an ulcerative colitis flare and 1 case of cavernous sinus thrombosis. Both cases required hospitalizations.

^d^Hospitalization and drug discontinuation were necessary for this event.

## Discussion

This is the first real-world experience study to report the effectiveness and safety of UST specifically in a Latin American population. The study focused on a group of mainly refractory UC patients, with a majority (80%) having prior biologic experience. These patients had a long disease duration (approximately 10 years), and 75% had pancolitis, with a baseline Mayo endoscopic subscore of 3 in 72.0% of cases. The co-primary endpoints of clinical remission at 1 year and endoscopic remission within 1 year were achieved by 50% and 36% of patients, respectively. In accordance with previously published real-world data, UST treatment was associated with improvements in clinical and biochemical parameters, along with high rates of drug persistence, low rates of colectomy, and a favorable safety profile.

The performance of a drug in real-world settings can differ from pivotal trials for various reasons. Pivotal trials often employ strict inclusion and exclusion criteria to ensure homogeneity and minimize confounding factors, while real-world settings encompass a broader spectrum of patients with varying disease severity, comorbidities, and concurrent medications. As an example, the UNIFI trial was the first registered trial to enroll UC patients who had previously failed more than 1 biologic therapy with different mechanisms of action.^18^ Among the 961 patients included, 51.1% had prior exposure to ≥1 biologic agent, and 16.6% had been exposed to both a TNF antagonist and vedolizumab. In this predominantly refractory population, the rate of clinical response at weeks 8 and 16 for patients receiving the labeled 6 mg/kg induction dose was 61.8% and 77.6%, respectively. Conversely, we demonstrated a clinical response rate at weeks 12–16 of 36% using an NRI analysis. We believe that the heterogeneity of patients in our cohort and the refractoriness of our population may have influenced early drug response. In our study, 50.0% of patients achieved the primary endpoint of clinical remission at 1 year, and 36.0% of patients achieved endoscopic remission within 1 year. The slightly higher clinical remission rates observed in our study could possibly be attributed to less strict criteria in definitions or our outcomes, in comparison to a registration clinical trial.

It’s important to note that real-world evidence complements pivotal trials by providing insights into a drug’s performance in routine clinical practice. While pivotal trials establish initial efficacy and short-term safety, real-world studies offer valuable information on long-term effectiveness, long-term safety profiles, treatment patterns, and patient outcomes in diverse populations. UST’s performance has been assessed in several real-life studies.

One study conducted by Hong et al.^16^ at 2 tertiary IBD centers in the USA aimed to evaluate the real-world effectiveness and safety of UST in patients with moderately to severely active UC. After 12 months, 45% and 35% of patients achieved clinical and corticosteroid-free remission, respectively. Interestingly, remission rates were quite similar to those observed at 3 months (42.6% and 35%, respectively), demonstrating sustained remission with UST in those who initially responded to the drug. Similarly, data from the GETAID multicenter real-world induction and maintenance studies also demonstrated sustained rates of clinical and steroid-free remission over time (39.8% and 35% at weeks 12–16, and 34% and 32% at week 52.^15^ In our cohort, clinical and steroid-free remissions were observed in 50% and 46.5% of patients at 1 year. In our sample, 20% of the patients were biologically naïve, 62% had previously been exposed to anti-TNFs, and 54% to vedolizumab, while 99% of patients were previously exposed to anti-TNF and 85% to vedolizumab in the GETAID cohort. Accordingly, 92% of patients had prior exposure to biologics and/or tofacitinib in the American cohort. It is also important to mention that local physicians’ practices in corticosteroid prescription for UC patients may vary based on factors such as regional guidelines, physician preferences, and patient characteristics, which may substantially impact steroid-free remission rates. Only a few real-world studies have reported the effectiveness of UST in improving objective markers of inflammation. Notably, despite the limited sample size, there was no significant difference observed in the efficacy of UST in inducing clinical remission among patients with prior exposure to either anti-TNF drugs or vedolizumab. Additionally, when used as a second-line treatment, UST demonstrated similar effectiveness in terms of endoscopic response, endoscopic remission, clinical response, and biochemical response/remission between patients previously exposed to vedolizumab or anti-TNF drugs. This finding suggests that UST may exhibit efficacy even in individuals with a history of exposure to biologics with distinct mechanisms of action.

A recent systematic review and meta-analyses by Taxonera et al.^19^ summarized the evidence on the real-world outcomes of UST in UC. The pooled rate of endoscopic improvement at weeks 12–16 was 29.9% (95% CI, 21.1–38.7; 91 patients; 2 studies). At month 12, 58.2% of patients (95% CI, 39.9–76.5) (80 patients) had endoscopic improvement and 26% of patients (95% CI, 10.4–41.6) achieved mucosal healing (40 patients; 2 studies). In our study, we reported endoscopic response and remission rates of 68% and 36%, respectively. Notably, we used the more stringent definition for endoscopic remission (endoscopic Mayo subscore of 0), as recommended by the Selecting Therapeutic Targets in Inflammatory Bowel Disease II (STRIDE II).^20^ Even though the rates of endoscopic improvement and mucosal healing match the data from the pivotal UNIFI trial, the reported results should be interpreted with caution since only a small proportion of patients underwent endoscopic evaluation in the aforementioned studies. The recent UNIFI long-term extension results have reassured UST treatment’s favorable safety profile up to 5 years of treatment.^21^ In our study, UST treatment was well tolerated, and no new safety signals were observed. Treatment discontinuations were mostly attributed to a lack of response or disease progression. Overall, 27 AEs were reported during the follow-up, with 16 of them classified as SAEs. Ten patients (20.0%) required hospitalization, and 3 patients (6.0%) underwent colectomy. No deaths were observed. Our results are consistent with those previously reported for UST.^12,14,15,22^

A total of 9 patients (18%) discontinued UST over time. The primary reasons for discontinuation were PNR in 7 patients (14%) and loss of response in 2 patients (4%) over a mean follow-up period of 19.2 months (ranging from 2 to 72 months). The cumulative probability of UST treatment persistence at the end of 2 years was 73.7%. In the meta-analysis conducted by Taxonera et al., the pooled rate of persistence with UST at month 12 was 73.3% (95% CI, 64.6–82) across 9 studies that included 634 patients.^19^ However, there was substantial between-study heterogeneity at this time point (I2 = 85%, Cochrane’s *Q* test *P* = .001). It’s important to note that most of these studies used per-protocol analyses instead of more stringent analyses, such as the NRI method.

Dose escalation of UST therapy to 4-weekly intervals was observed in 11 patients (22.0%) in our cohort. The proportion of patients with UC who regained biological and/or clinical response after increasing to a 4-weekly regimen was 54.5%. This rate was lower than that reported by Amiot et al.^15^ and Hong et al.,^16^ but similar to the rate reported in the Spanish real-life cohort.^11^ The need for dose intensification in our study was very similar to the recently reported need for dose optimization of UST in CD.^23^ In the present study, 10 patients (20.0%) were hospitalized, and 3 (6.0%) underwent colectomy during the follow-up. The role of biologics as disease-modifying agents in the natural course of UC has long been recognized.^24^ However, in the case of refractory patients, it is possible that UST treatment was maintained because there were no other available drugs to avoid surgery during the study period. Therefore, it is important to emphasize that the reluctance to recommend surgery may have deleterious consequences, as chronic mucosal inflammation can increase the risk of dysplasia and cancer.

This study is associated with some limitations that need to be discussed. First, it was a retrospective study with a noncontrolled design, which may result in patient dropouts, missing data, and some selection bias. Second, the number of patients included is somewhat small; however, it reflects the fact that the medication is not widely available in Brazil due to a lack of reimbursement. Third, the optimization of treatment with UST was done at the physician’s discretion and not according to a standardized protocol. The rate of AEs reported in our cohort was relatively low. However, due to the retrospective nature of the study, it could be argued that many of the adverse events were mild, were not reported by the patients, or did not require specific treatment, and therefore, were not entered into the medical records during follow-up appointments. Despite these limitations, our retrospective observational cohort has some strength and highlights the long-term effectiveness and safety of UST in UC patients from newly industrialized countries such as Brazil, where the prevalence of IBD has been increasing progressively in recent years.^25^ Additionally, in the current study, information on the patients was obtained through a structured questionnaire, with data collection standardization by the researchers. Moreover, we used objective clinical, biochemical, and endoscopic parameters commonly reported in real-world observational studies to describe therapy-induced meaningful objective outcomes in UC patients. Finally, future studies that include a broader population of patients with refractory UC treated with UST and with a longer follow-up duration are required to confirm outcomes over an extended period.

## Conclusions

In accordance with previously published real-world data, this multicenter real-world cohort of difficult-to-treat UC patients showed that UST was associated with improvements in clinical, biochemical, and endoscopic outcomes. The safety profile was consistent with previous studies.

## Data Availability

The full data of this study are available upon request and approval of central IRB.

## References

[1] Gros B , Kaplan GG. Ulcerative colitis in adults: a review. JAMA. 2023;330(10):951–965. doi:10.1001/jama.2023.15389 37698559

[2] Parra RS , Chebli JMF, Amarante HMBS, et al. Quality of life, work productivity impairment and healthcare resources in inflammatory bowel diseases in Brazil. World J Gastroenterol. 2019;25(38):5862–5882. doi:10.3748/wjg.v25.i38.5862 31636478 PMC6801193

[3] Dai N , Haidar O, Askari A, Segal JP. Colectomy rates in ulcerative colitis: a systematic review and meta-analysis. Dig Liver Dis. 2023;55(1):13–20. doi:10.1016/j.dld.2022.08.039 36180365

[4] da Costa Ferreira S , Otoboni Aprile LR, Serafim Parra R, et al. Factors predictive of proximal disease extension and clinical course of patients initially diagnosed with ulcerative proctitis in an IBD referral center. Turk J Gastroenterol. 2022;33(4):320–328. doi:10.5152/tjg.2022.21124 35550540 PMC9153924

[5] Tsai L , Ma C, Dulai PS, et al. Contemporary risk of surgery in patients with ulcerative colitis and Crohn’s disease: a meta-analysis of population-based cohorts. Clin Gastroenterol Hepatol. 2021;19(10):2031–2045.e11. doi:10.1016/j.cgh.2020.10.039 33127595 PMC8934200

[6] Vilela EG , Rocha HC, Moraes AC, et al. Inflammatory bowel disease care in Brazil: how it is performed, obstacles and demands from the physicians’ perspective. Arq Gastroenterol. 2020;57(4):416–427. doi:10.1590/S0004-2803.202000000-77 33331475

[7] Parra RS , da Costa Ferreira S, Machado VF, et al. Access to high-cost biological agents: perceptions of Brazilian patients with inflammatory bowel diseases. J Clin Med. 2023;12(7):2672. doi:10.3390/jcm12072672 37048755 PMC10095198

[8] Ustekinumab (Stelara) for Crohn’s disease. Med Lett Drugs Ther. 2017;59(1511):5–6. 28026835

[9] Efficacy of ustekinumab for ulcerative colitis through 4 years: final clinical and endoscopy outcomes from the UNIFI long-term extension. Gastroenterol Hepatol (N Y). 2023;19(4 Suppl 1):8. PMC10498105 37711661

[10] Ha C , Ullman TA, Siegel CA, Kornbluth A. Patients enrolled in randomized controlled trials do not represent the inflammatory bowel disease patient population. Clin Gastroenterol Hepatol. 2012;10(9):1002–1007; quiz e78. doi:10.1016/j.cgh.2012.02.004 22343692

[11] Chaparro M , Garre A, Iborra M, et al. Effectiveness and safety of ustekinumab in ulcerative colitis: real-world evidence from the ENEIDA Registry. J Crohns Colitis. 2021;15(11):1846–1851. doi:10.1093/ecco-jcc/jjab070 33860795 PMC8083263

[12] Thunberg J , Björkqvist O, Hedin CRH, et al.; SWIBREG Study Group. Ustekinumab treatment in ulcerative colitis: real-world data from the Swedish inflammatory bowel disease quality register. United Eur Gastroenterol J. 2022;10(7):631–639. doi:10.1002/ueg2.12275 PMC9486503 35834389

[13] Chiappetta MF , Viola A, Mastronardi M, et al. One-year effectiveness and safety of ustekinumab in ulcerative colitis: a multicenter real-world study from Italy. Expert Opin Biol Ther. 2021;21(11):1483–1489. doi:10.1080/14712598.2021.1981855 34521307

[14] Fumery M , Filippi J, Abitbol V, et al. Effectiveness and safety of ustekinumab maintenance therapy in 103 patients with ulcerative colitis: a GETAID cohort study. Aliment Pharmacol Ther. 2021;54(7):944–951. doi:10.1111/apt.16544 34296456

[15] Amiot A , Filippi J, Abitbol V, et al.; UC-USK-GETAID Study Group. Effectiveness and safety of ustekinumab induction therapy for 103 patients with ulcerative colitis: a GETAID multicentre real-world cohort study. Aliment Pharmacol Ther. 2020;51(11):1039–1046. doi:10.1111/apt.15717 32291786

[16] Hong SJ , Krugliak Cleveland N, Akiyama S, et al. Real-world effectiveness and safety of ustekinumab for ulcerative colitis from 2 tertiary IBD centers in the United States. Crohns Colitis 360. 2021;3(1):otab002. doi:10.1093/crocol/otab002 36777067 PMC9802405

[17] Satsangi J , Silverberg MS, Vermeire S, Colombel JF. The Montreal classification of inflammatory bowel disease: controversies, consensus, and implications. Gut. 2006;55(6):749–753. doi:10.1136/gut.2005.082909 16698746 PMC1856208

[18] Sands BE , Sandborn WJ, Panaccione R, et al.; UNIFI Study Group. Ustekinumab as induction and maintenance therapy for ulcerative colitis. N Engl J Med. 2019;381(13):1201–1214. doi:10.1056/NEJMoa1900750 31553833

[19] Taxonera C , Olivares D, López-García ON, Alba C. Meta-analysis: real-world effectiveness and safety of ustekinumab in patients with ulcerative colitis. Aliment Pharmacol Ther. 2023;57(6):610–619. doi:10.1111/apt.17386 36645145

[20] Turner D , Ricciuto A, Lewis A, et al.; International Organization for the Study of IBD. STRIDE-II: an update on the Selecting Therapeutic Targets in Inflammatory Bowel Disease (STRIDE) initiative of the International Organization for the Study of IBD (IOIBD): determining therapeutic goals for treat-to-target strategies in IBD. Gastroenterology. 2021;160(5):1570–1583. doi:10.1053/j.gastro.2020.12.031 33359090

[21] Sandborn WJ , Rebuck R, Wang Y, et al. Five-year efficacy and safety of ustekinumab treatment in Crohn’s disease: the IM-UNITI Trial. Clin Gastroenterol Hepatol. 2021;20(3):578–590.e4. doi:10.1016/j.cgh.2021.02.025 33618023 PMC8374005

[22] Honap S , Al-Hillawi L, Baillie S, et al. Ustekinumab for the treatment of moderate to severe ulcerative colitis: a multicentre UK cohort study. Frontline Gastroenterol. 2022;13(6):517–523. doi:10.1136/flgastro-2022-102168 36250172 PMC9555129

[23] Fumery M , Peyrin-Biroulet L, Nancey S, et al. Effectiveness and safety of ustekinumab intensification at 90 mg every four weeks in Crohn’s disease: a multicenter study. J Crohns Colitis. 2020;15(2):222–227. doi:10.1093/ecco-jcc/jjaa177 32898232

[24] Kaplan GG , Seow CH, Ghosh S, et al. Decreasing colectomy rates for ulcerative colitis: a population-based time trend study. Am J Gastroenterol. 2012;107(12):1879–1887. doi:10.1038/ajg.2012.333 23165448

[25] Quaresma AB , Damiao AOMC, Coy CSR, et al. Temporal trends in the epidemiology of inflammatory bowel diseases in the public healthcare system in Brazil: a large population-based study. Lancet Reg Health Americas 2022;13:100298. doi:10.1016/j.lana.2022.100298 36777324 PMC9903988

